# LINC01272 Suppressed Cell Multiplication and Induced Apoptosis Via Regulating MiR-7-5p/CRLS1 Axis in Lung Cancer

**DOI:** 10.4014/jmb.2102.02010

**Published:** 2021-05-27

**Authors:** Xuan Ma, Yang Liu, Hao Tian, Bo Zhang, Meiling Wang, Xia Gao

**Affiliations:** 1 Cancer Surgery Center, the Second People’s Hospital of China Three Gorges University, No. 4 Tiyuchang Road, Yichang, Hubei Province 443000, P.R. China; 2 Department of Pediatrics, Yichang First People’s Hospital, 443000, P.R.China

**Keywords:** LINC01272, lung cancer, miR-7-5p/CRLS1, multiplication, apoptosis

## Abstract

LINC01272 is a long non-coding RNA (lncRNA) that has been considered as a biomarker for many diseases including lung squamous cell carcinoma. Here, we investigated the function and mechanism of LINC01272 on lung cancer (LC). The differential expression of LINC01272 in LC and normal samples was analyzed by GEPIA based on the data from TCGA-LUAD database, as survival prognosis was analyzed through Kaplan-Meier Plotter. LINC01272 overexpression plasmid and miR-7-5p mimic were transfected into A549 and PC-9 cells. LINC01272, miR-7-5p and cardiolipin synthase 1 (CRLS1) mRNA expression was measured by quantitative reverse transcription-polymerase chain reaction. Cell viability was detected through MTT assay. Cell multiplication was evaluated by cell formation assay. Cell apoptosis was assessed through flow cytometry assay. Through bioinformatics, the target miRNA of LINC01272 and downstream genes of miR-7-5p were predicted. The targeting relationship was tested by dual luciferase reporter analysis. CRLS1, B-cell lymphoma-2 (Bcl-2), BCL2-associated X (Bax) and cleaved caspase-3 protein levels were detected through western blot. LINC01272 was downregulated in LC and low LINC01272 expression had poor prognosis. In A549 and PC-9 cells, LINC01272 inhibited cell viability and multiplication and induced apoptosis. LINC01272 negatively regulated miR-7-5p and CRLS1 was a target of miR-7-5p. MiR-7-5p reversed the effect of LINC01272 on viability, multiplication, apoptosis and expression of miR-7-5p and CRLS1 as well as apoptosis-related factors (Bcl-2, Bax and cleaved caspase-3). LINC01272 suppressed cell multiplication and induced apoptosis via regulating the miR-7-5p/CRLS1 axis in LC.

## Introduction

Lung cancer (LC) including non-small cell lung cancer (NSCLC) and small cell lung cancer (SCLC) is a most frequent malignant tumor around the world [5, 25]. According to cancer statistics, LC ranks 2^nd^ in the morbidity rate of malignancies and has become the No. 1 leading cause of cancer-associated mortality for both sexes, as 228,820 new cases as well as 135,720 deaths in total are estimated to have occurred in 2020 [33]. Smoking, viral infection, ionizing radiation, genetics and environmental factors like air pollution have been reported to intimately connect with increased risk of LC incidence [6, 24, 28, 34]. In spite of great progress made in diagnostic assay, most patients with LC are classified in an advanced stage at initial diagnosis [27]. Moreover, though tremendous effort has been exerted among LC treatments comprising surgery, chemotherapy, radiotherapy, immunotherapy and target therapy, relapse and resistance still happen frequently, leaving a poor prognosis with a 5-year overall survival rate of only 4-17% [4, 15, 25, 37]. Thus, studies on LC and its specific mechanisms are urgently needed to identify more capable biomarkers for diagnosis and prognosis as well as to find novel therapeutic targets for LC.

Possessing more than 200 nucleotides in length without protein-coding potential, long non-coding RNA (lncRNA) belongs to the big group of non-coding RNA, which has been misunderstood as “transcriptional noise” or “dark matter” for a long time [3, 21, 36]. In recent years, emerging evidence has gradually revealed the crucial regulatory role of lncRNA in gene expression by transcriptional/post-transcriptional regulation, genomic imprinting and chromatin modification [12, 19, 36]. In addition, dysregulation of lncRNA has been proved to be implicated in pathological processes of various diseases, like malignant tumors, thereby making lncRNA a promising target for the treatment of certain diseases [3, 9, 23]. Previous reports have validated the involvement of a lncRNA, LINC01272, in multiple diseases such as inflammatory bowel disease, Crohn’s disease and cardiovascular disease [13, 17, 36]. What’s more, LINC01272 advances cell metastasis through epithelial-mesenchymal transition in gastric cancer and its abnormal expression is also observed in lung squamous cell carcinoma [7, 23]. However, the specific effect of LINC01272 on LC and its mechanism of action have not yet been elucidated thus far.

More characterized and well-studied than lncRNA, microRNA (miRNA, miR) is another member of the non-coding RNA family, which plays a fundamental part in biological processes [30, 26, 8]. Former studies have shown that the interaction existing between miRNA and lncRNA can impact cell behavior [35]. Through bioinformatics, miR-7-5p was predicted to be a potential target of LINC01272. Hence, we supposed that LINC01272 might affect LC development via miR-7-5p.

## Materials and Methods

### Ethics Statement

The LC tissues were acquired from 47 patients with the disease in the Second People’s Hospital of China Three Gorges University from September 2017 to December 2019. All patients signed informed consent, agreeing that their tissues would be applied to clinical research. The clinical trial program was reviewed and approved by the Ethics Committee of the Second People’s Hospital of China Three Gorges University (TGH20170905810).

### Cell Culture

Human normal bronchial epithelial cell line BEAS-2B and LC cell lines Calu3, A549 and HCC827 were bought from American Type Culture Collection (ATCC; ATCC CRL-9609, ATCC HTB-55, ATCC CCL-185, ATCC CRL-2868, Rockefeller, MD, USA). A separate LC cell line, PC-9, was obtained from Shanghai Yaji Biological Technology Co., Ltd. (YS319C, China, http://www.yajimall.com/). BEAS-2B was incubated in bronchial epithelial growth medium (BEGM; CC-3170, Lonza/Clonetics Corporation, USA) in which we removed the Gentamycin-Amphotericin B mix (GA-1000). Calu3 cells were cultured in Eaglés Minimum Essential Medium (EMEM; ATCC 30-2003, ATCC, USA) while A549 cells were incubated in Kaighn's Modification of Ham's F-12 (F-12K) Medium (ATCC 30-2004). PC-9 and HCC827 cells were cultured in Roswell Park Memorial Institute (RPMI)-1640 medium (ATCC 30-2001, ATCC). All media except BEGM were supplemented with 10% fetal bovine serum (FBS; ATCC 30-2020, USA) and 1% Penicillin-Streptomycin Solution (ATCC 30-2300, ATCC). All cells were incubated in a humidified atmosphere at 37°C with 5% CO_2_.

### Bioinformatics

Gene Expression Profiling Interactive Analysis (GEPIA; http://gepia.cancer-pku.cn/) was utilized for analysis of the differentially expressed LINC01272 between LC (*n* = 486) and normal tissues (*n* = 338) based on the data from The Cancer Genome Atlas Lung Adenocarcinoma (TCGA-LUAD) database. The survival prognosis was analyzed through Kaplan-Meier Plotter (www.kmplot.com). The downstream miRNAs of LINC01272 were predicted by LncRNASNP2 (http://bioinfo.life.hust.edu.cn/lncRNASNP/#!/) in combination with analysis of TCGA-LUAD database. The StarBase (http://starbase.sysu.edu.cn/starbase2/), miRDB (http://mirdb.org/), TargetScan (http://www.targetscan.org/vert_72/), miRWalk (http://mirwalk.umm.uni-heidelberg.de/), miRTarbase (http://mirtarbase.cuhk.edu.cn/php/index.php) and TCGA-LUAD databases were adopted to select downstream target genes of miR-7-5p.

### Cell Transfection

The overexpression vector (pcDNA3.1/+vector; V79020, Invitrogen, Thermo Fisher Scientific, USA) carrying LINC01272 gene and its negative control (NC; empty vector pcDNA3.1/+vector), miR-7-5p mimic (M; miR10000252-1-5, Guangzhou RiboBio Co., Ltd., China) and mimic control (MC; miR1N0000001-1-10, Guangzhou RiboBio Co., Ltd.) were transfected into A549 and PC-9 cells with Lipo8000 Transfection Reagent (C0533, Beyotime Biotechnology, China). First, cells were trypsinized (C0205, Beyotime Biotechnology), seeded in 6-well plates at 4 × 10^5^ cells/well and cultured until 70-80% fusion. Then, 2.5 μg plasmid DNA was diluted into 125 μl Opti-MEM medium (31985062, Thermo Fisher Scientific), following which Lipofectamine 8000 (4 μl) was added. Finally, 125 μl DNA-reagent mixture was added to each well and cells were incubated at 37°C for 24 or 48 h.

### Quantitative Reverse Transcription-Polymerase Chain Reaction (qRT-PCR)

Tissues were ground in a homogenizer (D-500, Wiggens, Straubenhardt, Germany, http://www.wiggens.com/), subsequent to which total mRNA and miRNA of tissues and cells were isolated using RNAiso Plus (Trizol) (9109, Takara Biomedical Technology Co., Ltd., China) and RNAiso for Small RNA (9753A, Takara Biomedical Technology Co., Ltd.), respectively. PrimeScript RT Reagent Kit with gDNA Eraser (Perfect Real Time) (RR047A, Takara Biomedical Technology Co., Ltd.) was adopted to synthesized cDNA of mRNA and miRNA through reverse transcription. The cDNA amplification was traced by TB Green Fast qPCR Mix (RR430S, Takara Biomedical Technology Co., Ltd.) in an Applied Biosystems 7500 Fast Real-Time PCR System (ABI, Thermo Fisher Scientific) under a condition as follows: predenaturation at 95°C for 30 s, followed by 40 cycles of 95°C for 3 s and 60°C for 15 s. The primer sequences (Guangzhou RiboBio Co., Ltd.) were presented in Table 1. GAPDH and U6 served as endogenous controls of mRNA and miRNA, respectively, as data were calculated by 2^-ΔΔCT^ relative quantification method [29].

### MTT Assay

Cell viability was detected through MTT Cell Multiplication and Cytotoxicity Assay Kit (C0009S, Beyotime Biotechnology). After trypsin digestion, cells were seeded at a concentration of 2 × 10^3^ cells/well in 96-well plates, and cultured with 5% CO_2_ at 37°C for 24 h. Subsequently, 10 μl/well MTT solution was added and cells continued to be incubated at 37°C for another 4 h. With MTT solution discarded, a 100-μl solution for dissolving formazan was added into each well for 10 min again to dissolve the crystal completely. A Cytation Hybrid Multi-Mode microplate reader (BioTek Instruments, Inc. USA) was employed to measure the absorbance (A) at 570nm. Relative cell viability (%) = (A of treated group/A of negative control group) × 100.

### Colony Formation Assay

Trypsinized and seeded in 6-well plates at 500 cells/well, cells were incubated with medium changed every two days until colonies were formed. After removal of medium, cells were fixed with 4% paraformaldehyde (1 ml/well; P0099, Beyotime Biotechnology, China) at room temperature for 20 min, subsequent to which cells were rinsed twice by phosphate-buffered saline (PBS) for 2 min and Crystal Violet Staining Solution (200 μl/well; C0121, Beyotime Biotechnology) was added for 10 min to stain cells. After being washed with PBS, cells were observed and counted under a Leica DM1000 biological microscope (Leica Microsystems, Germany, https://www.leica-microsystems.com.cn/cn/).

### Flow Cytometry Assay

The Annexin V-FITC/PI Apoptosis Detection Kit (E606336, Sangon Biotech Co., Ltd., China) was applied for cell apoptosis assessment. Briefly, 4 × binding buffer was diluted into 1 × binding buffer, in which cells were suspended and adjusted to a density of 4 × 10^5^ cells/ml. Next, 5 μl Annexin V-FITC was added into 195 μl cell suspension and mixed well in a dark room, followed with incubation for 10 min at room temperature. Then, cells were rinsed using 1 × binding buffer (200 μl) and centrifuged for 5 min at 1,000 ×*g*. Supernatant removed, cells were resuspended in 190 μl binding buffer (1 ×) and 10 μl PI added. Cell apoptosis was analyzed using a flow cytometer (Multiskan FC, Thermo Fisher Scientific).

### Dual Luciferase Reporter Analysis

The 3’-untranslational region (UTR) sequence of human LINC01272/ cardiolipin synthase 1 (CRLS1) was inserted into the pmirGLO vectors (E1330, Promega, USA) to establish the wild-type LINC01272 (LINC01272-WT, 5’-GAAGTCTCTTCCCTGGTCTTCCA-3’)/wild-type CRLS1 (CRLS1-WT, 5’-AGTTCATTTTCGGTA GTCTTCCA-3’) while the other pmirGLO vector containing mutative LINC01272 3’-UTR sequence (LINC01272-MUT, 5’-GAAGTCTCTTCCCAGCTCTCCCA-3’)/CRLS1 3’-UTR sequence (CRLS1-MUT, 5’-AGTTCATTTTCGGTAGTCATCAA-3’) in the putative miR-7-5p seed region served as a negative control, respectively. Cells were co-transfected with LINC01272-WT/CRLS1-WT or LINC01272-MUT/CRLS1-MUT and miR-7-5p mimic for 24 h. A Dual-Luciferase Reporter Assay System (E1910, Promega) was utilized to analyze the luciferase activity.

### Western Blot

Total protein was extracted by Radio Immunoprecipitation Assay (RIPA) buffer (R0020, Beijing Solarbio Science & Technology Co., Ltd., China), followed by centrifugation at 12,000 ×*g* for 10 min at 4°C. With supernatant collected, total protein concentration was tested through BCA Protein Assay Kit (PC0020, Beijing Solarbio Science & Technology Co., Ltd.). Equal amounts of protein and ColorMixed Protein Marker (11-180 kDa) (PR1910, Beijing Solarbio Science & Technology Co., Ltd.) were separated by 15% SDS (P0012A, Beyotime Biotechnology) polyacrylamide gel electrophoresis (SDS-PAGE). Then, protein was transferred to PVDF membranes (88585, Thermo Fisher Scientific) blocked in 5% bovine serum albumin (BSA; SW3015, Beijing Solarbio Science & Technology Co., Ltd.) blocking buffer for 1 h and cultured at 4°C with rabbit anti-CRLS1 (1:800; ab156882, Abcam, USA), rabbit anti-B-cell lymphoma-2 (Bcl-2; 1:500; ab59348, Abcam, USA), rabbit anti-Bcl2-associated X (Bax; 1:1000; ab32503, Abcam), rabbit anti-cleaved caspase-3 (1:500; ab2302, Abcam) and mouse anti-GAPDH (1:1000; ab8245, Abcam) overnight. Next, membranes were washed four times with Tris-buffered saline containing Tween 20 (TBST; T1085, Beijing Solarbio Science & Technology Co., Ltd.), subsequent to which cells were incubated with corresponding secondary antibodies conjugated to anti-rabbit IgG H&L (ab6721, 1:5000; Abcam) and anti-mouse IgG H&L (ab6728, 1:5000; Abcam) at room temperature for 1 h. After being rinsed five times with TBST for 5 min, membranes were mixed with Electrochemiluminescence (ECL) Western Blotting Substrate (PE0010, Beijing Solarbio Science & Technology Co., Ltd.) in Tanon 5200 Imaging System (Shanghai Tanon Technology Co., Ltd., China) to visualize protein. Finally, the result was analyzed through Image J software, version 1.48 (National Institutes of Health, USA).

### Statistical Analysis

GraphPad Prism 8.0 (GraphPad Software Inc., USA) was adopted for analysis of data which was performed as the means ± SD. All experiments were carried out three times at least. Student’s t-test was used to compare the significant difference between two groups while multiple groups were compared by one-way ANOVA with Dunnett’s or Tukey’s post hoc test. A Venn diagram was adopted to identify the intersecting genes. *p* < 0.05 was regarded as an indicator of statistically significant difference.

## Results

### LINC01272 Was Downregulated in LC and Low LINC01272 Expression Was Correlated with Poor Prognosis

**Fig. 1:**
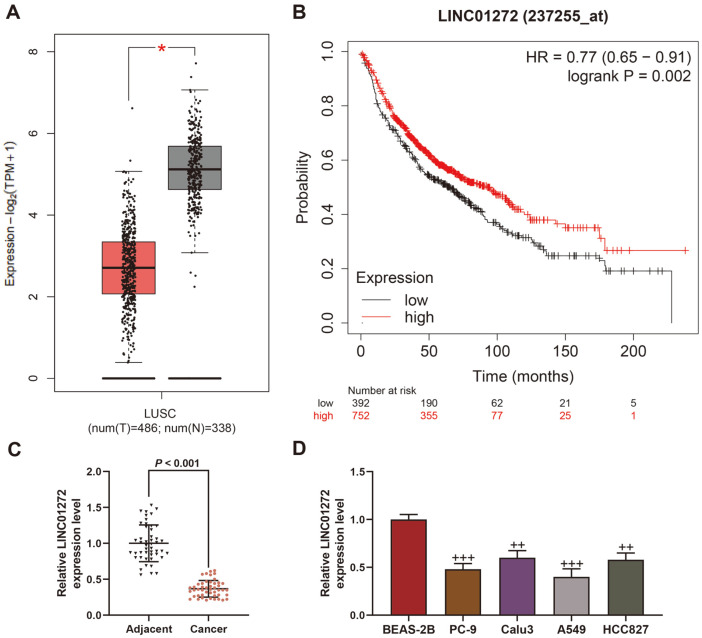
LINC01272 was downregulated in LC and low LINC01272 expression had poor prognosis. (**A**) The differential expression of LINC01272 between LC (*n* = 486) and normal samples (*n* = 338) was analyzed through GEPIA based on the data from TCGA-LUAD database. (**B**) The survival prognosis in low and high LINC01272 expression was analyzed through Kaplan-Meier Plotter. (**C**) Relative LINC01272 expression between LC and adjacent normal tissues was detected by qRT-PCR. (**D**) Relative LINC01272 expression between LC (PC-9, Calu3, A549 and HCC827) and normal cell lines (BEAS-2B) was detected by qRT-PCR. GAPDH was a loading control. ^++^*p* < 0.01, ^+++^*p* < 0.001 vs. BEAS-2B cells. All experiments were repeated independently at least three times. Data were performed as the means ± SD. Abbreviations: LC, lung cancer; GEPIA, Gene Expression Profiling Interactive Analysis; TCGA-LUAD, The Cancer Genome Atlas Lung Adenocarcinoma; qRT-PCR, quantitative reverse transcription-polymerase chain reaction; GAPDH, glyceraldehyde-3-phosphate dehydrogenase.

Through GEPIA, LINC01272 was reported to be downregulated in LC samples in comparison with normal samples (Fig. 1A, *p* < 0.05). And the analysis of the relationship between LINC01272 and survival prognosis showed that low expression level of LINC01272 and poor prognosis are correlated (Fig. 1B, *p* = 0.002). Similar to the consequence of GEPIA analysis in LC and normal samples, when contrasted with normal tissues and cells, LC tissue and cells presented lower LINC01272 level (Figs. 1C and 1D, *p* < 0.01). Those findings implicated that LINC01272 might become a prognostic biomarker for LC.

### LINC01272 Inhibited LC Cell Viability and Multiplication While Inducing Apoptosis

**Fig. 2:**
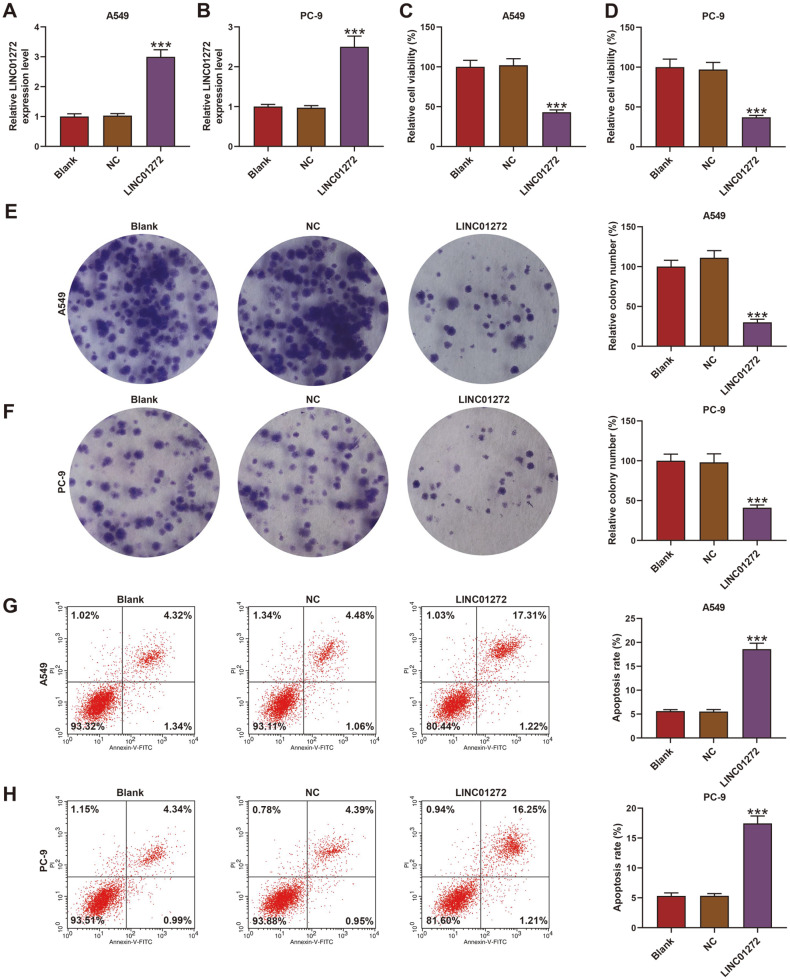
LINC01272 inhibited LC cell viability and multiplication while inducing apoptosis. (**A** and **B**) Relative LINC01272 expression of A549 (**A**) and PC-9 (**B**) cells was tested by qRT-PCR after transfection of LINC01272 overexpression plasmid. GAPDH was a loading control. (**C** and **D**) Relative cell viability of A549 (**C**) and PC-9 (**D**) cells was assessed by MTT assay after transfection of LINC01272 overexpression plasmid. (**E** and **F**) Relative colony number of A549 (**E**) and PC-9 (**F**) cells was evaluated through colony formation assay after transfection of LINC01272 overexpression plasmid. (**G** and **H**) Apoptosis rate of A549 (**G**) and PC-9 (**H**) cells was measured by Annexin V-FITC Apoptosis Detection Kit with flow cytometry after transfection of LINC01272 overexpression plasmid. ***p < 0.001 vs. NC group. All experiments were repeated independently at least three times. Data were performed as the means ± SD. Abbreviations: LC, lung cancer; qRT-PCR, quantitative reverse transcription-polymerase chain reaction; GAPDH, glyceraldehyde-3-phosphate dehydrogenase; NC, negative control for LINC01272 overexpression plasmid.

Owing to the lowest expression of LINC01272 exhibited in PC-9 and A549 cells among LC cell lines (Fig. 1D, *p* < 0.01), LINC01272 overexpression plasmid was transfected into those two cell lines. The transfection efficiency was tested by qRT-PCR, the result of which demonstrated that LINC01272 expression in A549 and PC-9 cells prominently increased after the transfection (Figs. 2A and 2B, *p* < 0.001), indicating the successful establishment of overexpressed LINC01272 model. During the MTT assay, we discovered that cell viability of A549 and PC-9 cells in LINC01272 group decreased in contrast with NC group (Figs. 2C and 2D, *p* < 0.001), as a similar outcome was acquired in the colony formation assay (Figs. 2E and 2F, *p* < 0.001). On the contrary, a significant rise of apoptosis rate in A549 and PC-9 cells transfected with LINC01272 overexpression plasmid was viewed when compared with cells transfected with NC (Figs. 2G and 2H, *p* < 0.001). These results implied that LINC01272 might fulfill a positive function against LC through inhibiting cell viability and multiplication while inducing apoptosis.

### MiR-7-5p Was Negatively Regulated by LINC01272

**Fig. 3:**
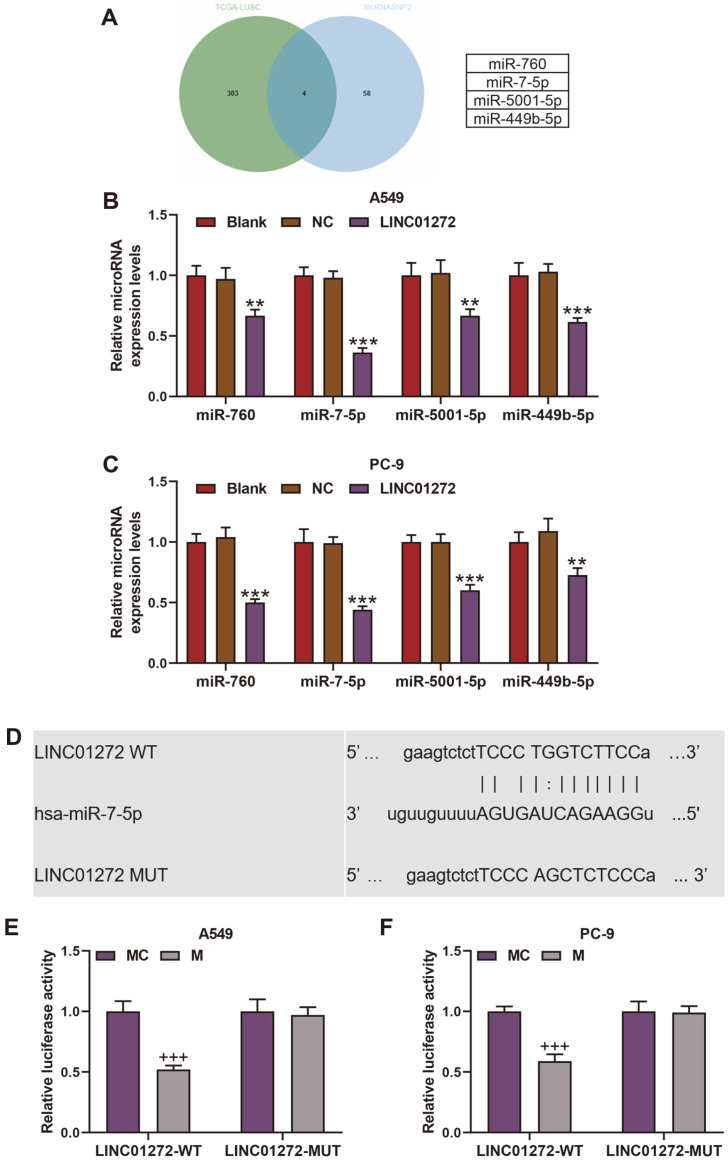
MiR-7-5p negatively correlated with LINC01272 in LC cells, and miR-7-5p was a target of LINC01272. (**A**) Venn diagram of potential target miRNAs from prediction of LncRNASNP2 and analysis based on TCGA-LUAD database. (**B** and **C**) Relative miR-760, miR-7-5p, miR-5001-5p and miR-449b-5p expression of A549 (**B**) and PC-9 (**C**) cells was detected by qRT-PCR after transfection of LINC01272 overexpression plasmid. U6 was a loading control. (**D**) The binding sites between LINC01272 and miR-7-5p. (**E** and **F**) Relative luciferase activity of A549 (**B**) and PC-9 (**C**) cells was tested by dual-luciferase reporter analysis after transfection of LINC01272-WT and miR-7-5p mimic. **p < 0.01, ***p < 0.001 vs. NC group; +++*p* < 0.001 vs. MC group. All experiments were repeated independently at least three times. Data were performed as the means ± SD. Abbreviations: LC, lung cancer; TCGA-LUAD, The Cancer Genome Atlas Lung Adenocarcinoma; qRT-PCR, quantitative reverse transcription-polymerase chain reaction; NC, negative control for LINC01272 overexpression plasmid; LINC01272- WT, wild-type LINC01272, MC, miR-7-5p mimic control.

Through bioinformatics, two groups of potential target miRNAs for LINC01272 were identified, in which 62 miRNAs from the prediction of LncRNASNP2 and 307 miRNAs from the analysis based on TCGA-LUAD database (Fig. 3A). The overlap in the Venn diagram among the two sets of miRNAs contained 4 miRNAs: miR-760, miR-7-5p, miR-5001-5p and miR-449b-5p (Fig. 3A). The qRT-PCR results showed that the levels of those four miRNAs in A549 and PC-9 cells of LINC01272 group were lower than NC group, among which the reduction of miR-7-5p was most obvious (Figs. 3B and 3C, *p* < 0.01). Moreover, LncRNASNP2 also predicted that LINC01272 had binding sites on miR-7-5p (Fig. 3D). And the result was confirmed by dual-luciferase reporter analysis which exhibited a prominent decline of luciferase activity in LINC01272-WT group after transfection of miR-7-5p mimic (Figs. 3E and 3F, *p* < 0.001), suggesting a negative correlation between LINC01272 and miR-7-5p.

### CRLS1 Functioned as a Target of miR-7-5p

**Fig. 4:**
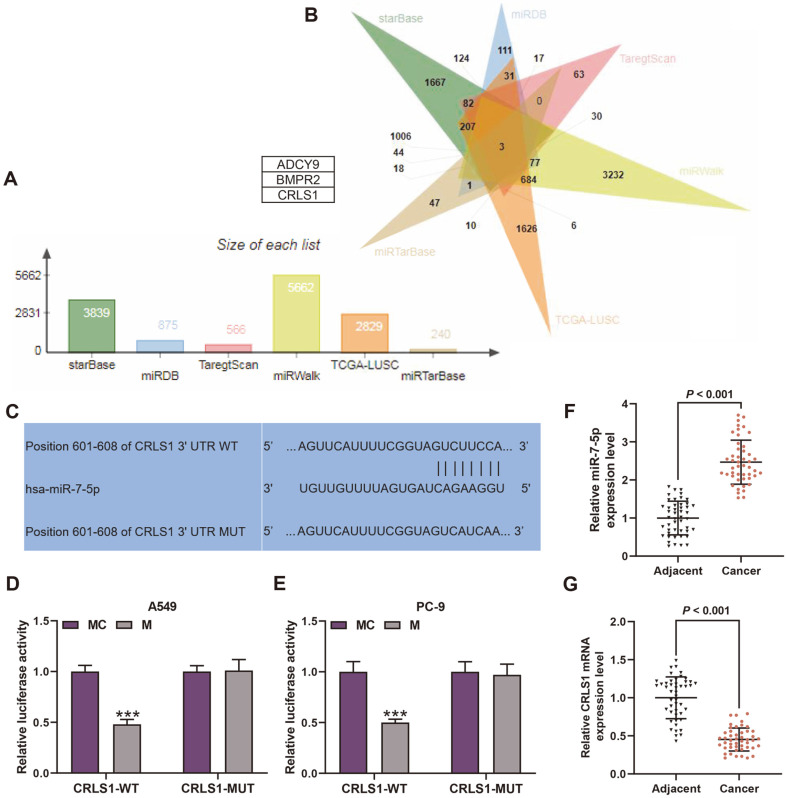
CRLS1 functioned as a target of miR-7-5p. (**A**) The number of potential target genes for miR-7-5p from StarBase, miRDB, TargetScan, miRWalk and miRTarbase and TCGA-LUAD database. (**B**) The Venn diagram of potential target genes for miR-7-5p. (**C**) The binding sites between miR-7-5p and CRLS1. (**D** and **E**) Relative luciferase activity of A549 (**D**) and PC-9 (**E**) cells was assessed through dual-luciferase reporter analysis after transfection of CRLS1-WT and miR-7-5p mimic. (**F**) Relative miR-7-5p expression between LC and adjacent normal tissues was evaluated by qRT-PCR. (**G**) Relative CRLS1 expression between LC and adjacent normal tissues was measured through qRT-PCR. ***p < 0.001 vs. MC group. All experiments were repeated independently at least three times. Data were performed as the means ± SD. Abbreviations: CRLS1, cardiolipin synthase 1; TCGA-LUAD, The Cancer Genome Atlas Lung Adenocarcinoma; CRLS1-WT, wild-type CRLS1; LC, lung cancer; qRT-PCR, quantitative reverse transcription-polymerase chain reaction; MC, miR-7-5p mimic control.

After screening the potential target genes of miR-7-5p, we acquired 3,839 genes from StarBase, 875 genes from miRDB, 566 genes from TargetScan, 5,662 genes from miRWalk, 240 genes from miRTarbase and 2,829 genes from TCGA-LUAD database (Fig. 4A). The Venn diagram revealed the overlap in the six sets of genes consisting of 3 genes: ADCY9, BMPR2 and CRLS1 (Fig. 4B). Due to the prediction that miR-7-5p closely bound to CRLS1 (Fig. 4C), dual-luciferase reporter analysis was utilized for verification. As a result, both A549 and PC-9 cells transfected with CRLS1-WT decreased luciferase activity after transfection with miR-7-5p in comparison with transfection with mimic control (Figs. 4D and 4E, *p* < 0.001) while no significant difference was observed in CRLS1-MUT groups (Figs. 4D and 4E). Furthermore, when compared with adjacent normal tissues, miR-7-5p expression elevated in LC cancer tissues and CRLS1 mRNA level reduced (Figs. 4F and 4G, *p* < 0.001).

### MiR-7-5p Mimic Reversed the Effect of LINC01272 on miR-7-5p and CRLS1 Expression of LC Cells

**Fig. 5:**
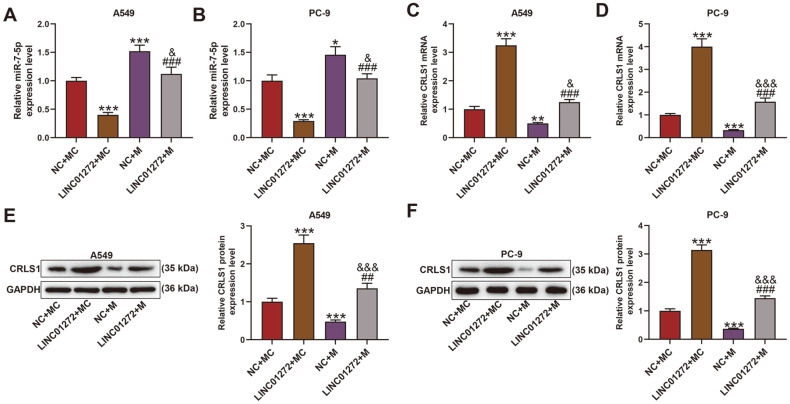
MiR-7-5p mimic reversed the effect of LINC01272 on miR-7-5p and CRLS1 expression of LC cells. (**A** and **B**) Relative miR-7-5p expression between of A549 (**A**) and PC-9 (**B**) cells was detected through qRT-PCR after transfection of miR-7-5p mimic and LINC01272 overexpression plasmid. U6 was a loading control. (**C** and **D**) Relative CRLS1 mRNA expression between of A549 (**C**) and PC-9 (**D**) cells was tested by qRT-PCR after transfection of miR-7-5p mimic and LINC01272 overexpression plasmid. GAPDH was a loading control. (**E** and **F**) Relative CRLS1 protein expression between of A549 (**E**) and PC-9 (**F**) cells was assessed through western blot after transfection of miR-7-5p mimic and LINC01272 overexpression plasmid. GAPDH was a loading control. *p < 0.05, **p < 0.01, ***p < 0.001 vs. NC+MC group; ^##^*p* < 0.01, ^###^*p* < 0.001 vs. LINC01272+MC group; ^&^*p* < 0.05, ^&&&^*p* < 0.001 vs. NC+M group. All experiments were repeated independently at least three times. Data were performed as the means ± SD. Abbreviations: CRLS1, cardiolipin synthase 1; LC, lung cancer; qRT-PCR, quantitative reverse transcription-polymerase chain reaction; GAPDH, glyceraldehyde-3-phosphate dehydrogenase; NC, negative control for LINC01272 overexpression plasmid; MC, miR-7-5p mimic control; M, miR-7-5p mimic.

After transfection of miR-7-5p mimic and LINC01272 overexpression plasmid, we found that miR-7-5p levels of A549 and PC-9 cells in NC+M group rose (Figs. 5A and 5B, *p* < 0.05) while they declined in LINC01272+MC group (Figs. 5A and 5B, *p* < 0.001) in contrast with NC+MC group, with miR-7-5p expression of LINC01272+MC group higher than LINC01272+MC group (Figs. 5A and 5B, *p* < 0.001) and lower than NC+M group (Figs. 5A and 5B, *p* < 0.05). Conversely, when compared with NC+MC group, both A549 and PC-9 cells performed higher CRLS1 mRNA and protein expression after transfection of LINC01272 (Figs. 5C-5F, *p* < 0.001) while a marked reduction was viewed in cells transfected with miR-7-5p mimic (Figs. 5C-5F, *p* < 0.01). In comparison with LINC01272+MC group, CRLS1 mRNA and protein expression of cells co-transfected with LINC01272 and miR-7-5p mimic downregulated (Figs. 5C-5F, *p* < 0.001), whereas they upregulated when contrasted with NC+M group (Figs. 5C-5F, *p* < 0.05).

### MiR-7-5p Mimic Reversed the Effect of LINC01272 on Viability, Multiplication, Apoptosis and Levels of Apoptosis-Related Factors in LC Cells

**Fig. 6:**
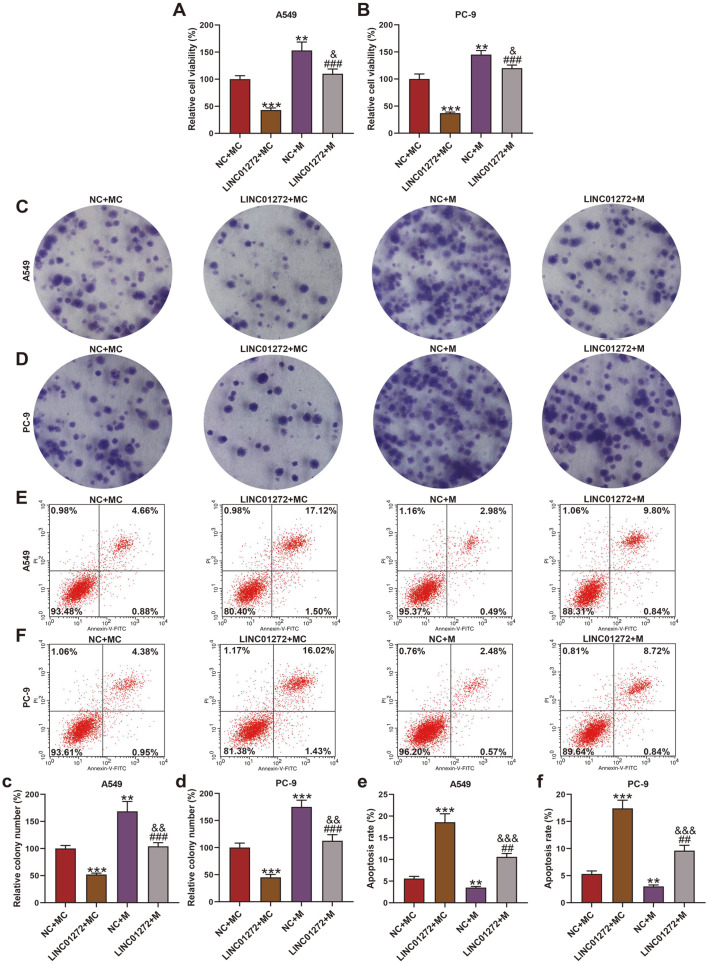
MiR-7-5p mimic reversed the effect of LINC01272 on LC cell viability, multiplication and apoptosis. (**A** and **B**) Relative cell viability of A549 (**A**) and PC-9 (**B**) cells was assessed by MTT assay after transfection of miR-7-5p mimic and LINC01272 overexpression plasmid. (**C** and **D**) Colony number of A549 (**C**) and PC-9 (**D**) cells was evaluated through colony formation assay after transfection of miR-7-5p mimic and LINC01272 overexpression plasmid. (**E** and **F**) Apoptosis rate of A549 (**E**) and PC-9 (**F**) cells was measured by Annexin V-FITC Apoptosis Detection Kit with flow cytometry after transfection of miR-7-5p mimic and LINC01272 overexpression plasmid. **p < 0.01, ***p < 0.001 vs. NC+MC group; ^##^*p* < 0.01, ^###^*p* < 0.001 vs. LINC01272+MC group; ^&^*p* < 0.05, ^&&^*p* < 0.01, ^&&&^*p* < 0.001 vs. NC+M group. All experiments were repeated independently at least three times. Data were performed as the means ± SD.

The MTT assay showed that the cell viability of A549 and PC-9 cells rose in NC+M group (Figs. 6A and 6B, *p* < 0.01) and decreased in LINC01272+MC group (Figs. 6A and 6B, *p* < 0.001) in contrast with NC+MC group, as cells in LINC01272+M group presented higher cell viability than LINC01272+MC group (Figs. 6A and 6B, *p* < 0.001) and lower than NC+M group (Figs. 6A and 6B, *p* < 0.05). Similarly, when compared with NC+MC group, cells transfected with LINC01272 overexpression plasmid formed fewer colonies (Figs. 6C and 6D, *p* < 0.001) while cells transfected with miR-7-5p mimic formed more colonies (Figs. 6C and 6D, *p* < 0.01), with the colony number of cells co-transfected with LINC01272 and miR-7-5p mimic higher than cells transfected with LINC01272 overexpression plasmid (Figs. 6C and 6D, *p* < 0.001) and lower than cells transfected with miR-7-5p mimic (Figs. 6C and 6D, *p* < 0.01). The opposite consequence was obtained during the cell apoptosis evaluation (Figs. 6E and 6F, *p* < 0.01). Through western blot, Bcl-2 level declined and Bax as well as cleaved caspase-3 expression elevated in A549 and PC-9 cells of LINC01272+MC group when contrasted with NC+MC group (Figs. S1A and S1B, *p* < 0.01), whereas NC+M group showed a contrary result (Figs. S1A and S1B, *p* < 0.01). And the Bcl-2 level of A549 and PC-9 cells in LINC01272+M group was higher than LINC01272+MC group (Figs. S1A and S1B, *p* < 0.01) and lower than NC+M group (Figs. S1A and S1B, *p* < 0.01) while Bax and cleaved caspase-3 expression in LINC01272+M group were lower than LINC01272+MC group (Figs. S1A and S1B, *p* < 0.01) and higher than NC+M group (Figs. S1A and S1B, *p* < 0.001). Those data indicated that LINC01272 promoted cell apoptosis and suppressed cell viability and multiplication through downregulating miR-7-5p in LC.

## Discussion

Although both the incidence and fatal rate related to LC have declined in the past few decades, LC is still the most common malignancy which is reported to bring about more deaths in 2017 than other top common cancers (colorectal, breast, prostate and brain cancers) combined [33]. It is believed that the identification of reliable biomarkers may provide a new key for LC diagnosis and prognosis as well as help to discover possible targets for LC treatment. Accumulating evidence has determined that lncRNAs, a research area highlighted in recent years, achieves diverse functions in the oncogenesis of LC, and is also regarded to offer an innovative platform for seeking feasible markers of LC [7, 18]. [23] LINC01272 was significantly upregulated in tissue and plasma samples from patients with inflammatory bowel disease, and it may be a potential diagnostic biomarkers for inflammatory bowel disease [36]. In addition, LINC01272 was upregulated in unstable atherosclerotic plaque, and knockdown of PELATON affects cellular functions associated with plaque progression [17]. LINC01272 expression was correlated with mucosal injury in Crohn's disease [13]. In our study, the analysis of bioinformatics illustrated that LINC01272 expression level significantly decreased in LC and low expression level of LINC01272 and poor prognosis are correlated, which was consistent with the report about the aberrant expression of lncRNA in lung squamous cell carcinoma [7]. And the results of qRT-PCR verified the analysis of differentially expressed LINC01272 between LC and normal tissues, which showed that LINC01272 was downregulated in LC tissues and cells. Those observations implicated a possibility of LINC01272 being a diagnostic and prognostic biomarker for LC. In addition, LINC01272 has been reported to promote migration and invasion of gastric cancer cells via EMT [23]. LINC01272 palys different roles in different cancers and these differences may be related to the tumor microenvironment.

Owing to the lower LINC01272 levels presented in A549 and PC-9 cells among LC cell lines, we transfected LINC01272 overexpression plasmid into the two cell lines to construct a model of overexpressed LINC01272 for a better and more obvious experimental effect, with qRT-PCR testing to see whether the transfection succeeded. Through our experiments, we discovered that the overexpression of LINC01272 repressed viability and multiplication while inducing apoptosis of LC cells, suggesting a protective function of LINC01272 against LC progression.

To explore the mechanism of LINC01272 on LC, we firstly located the target miRNAs of LINC01272 with the help of LncRNASNP2 prediction and TCGA-LUAD database analysis using the Venn diagram. Among four potential miRNAs (miR-760, miR-7-5p, miR-5001-5p, and miR-449b-5p), miR-7-5p expression was greatly downregulated after transfection of LINC01272 overexpression plasmid, with the binding sites predicted between LINC01272 and miR-7-5p, and dual-luciferase reporter analysis as well as upregulation of miR-7-5p in LC tissues proving that LINC01272 negatively targeted miR-7-5p in LC. Secondly, miR-7-5p mimic and LINC01272 overexpression plasmid were transfected into LC cells to further verify the mechanism whereby LINC01272 realized the effect on LC cells via miR-7-5p, as qRT-PCR was utilized for transfection efficiency test. Through MTT assay, colony formation assay and flow cytometry assay, we found that miR-7-5p advanced LC cell viability and multiplication while suppressing apoptosis and partly offsetting the influence of LINC01272 on LC.

Bcl-2 and Bax are two members of the Bcl-2 family and play a pivotal part in cellular apoptosis, fulfilling their functions in a converse way, with Bcl-2 an anti-apoptotic molecule and Bax a pro-apoptotic factor [2, 38]. Belonging to caspase family, caspase-3 has been confirmed as an executioner that induces cell apoptosis after turning into its active form “cleaved caspase-3” when cleaved by apoptotic stimuli [20, 22, 32]. The results of our study presented that miR-7-5p inhibited pro-apoptotic factors (Bax and cleaved caspase-3) and promoted anti-apoptotic molecule (Bcl-2) level, reversing the effect of LINC01272 on those apoptosis-associated protein expressions.

Thirdly, we searched the downstream target genes of miR-7-5p. Through a Venn diagram based on data from StarBase, miRDB, TargetScan, miRWalk, miRTarbase and TCGA-LUAD database, three genes were selected, among which CRLS1 was predicted to closely bind to miR-7-5p. The dual-luciferase reporter analysis validated the prediction and revealed that miR-7-5p inhibited CRLS1 expression of LC cells, as a downregulation of CRLS1 was performed in LC tissues. Cardiolipin is a kind of phospholipid widely existing in mitochondrial membrane, and has been determined to be necessary for the activation of several key mitochondrial membrane enzymes participating in mitochondrial respiratory chain components and synthesis of ATP [10, 14, 39]. Moreover, cardiolipin is considered to be implicated in cell apoptosis [11, 31]. CRLS1 is an enzyme adopted for catalysis during cardiolipin synthesis [16]. Former studies have demonstrated that anti-cardiolipin antibodies elevated thrombosis rate in breast cancer and CRLS1 was taken as a tumor suppressor of NSCLC [1, 10]. In this research, overexpressed LINC01272 promoted CRLS1 expression and reversed the CRLS1 downregulation induced by miR-7-5p. These results demonstrate that LINC01272 regulated miR-7-5p/CRLS1 axis in LC.

Considering all of the above data and analysis, this paper expounded the role of LINC01272 in LC and its specific mechanism, providing more comprehensive insights into the complex molecular mechanisms of LC and conducing to novel therapeutic strategies for LC. In the future, experiments in vivo will be conducted to validate the conclusion obtained in this study and we will continue to search for more target genes of LINC01272 and probe into their respective mechanisms through which LINC01272 impacts upon LC advancement.

In summary, the paper demonstrated that LINC01272 suppressed cell multiplication and induced apoptosis by sponging miR-7-5p in LC via upregulating CRLS1, which implies that LINC01272 might be a biomarker and therapeutic target for LC.

## Supplemental Materials

Supplementary data for this paper are available on-line only at http://jmb.or.kr.

## Figures and Tables

**Table 1:** Primer sequences used for quantitative reverse transcription-polymerase chain reaction (qRT-PCR).

Target gene	Primers, 5’-3’
LINC01272	
(Forward)	CCAAGGTCACGCAGCACAGTC
(Reverse)	GCAGAGATGAGCAGCAGTGGTG
miR-760	
(Forward)	GGGAGTCGTATCCAGTGCAA
(Reverse)	GTCGTATCCAGTGCGTGTCG
miR-7-5p	
(Forward)	GTTGTTGTCGTATCCAGTGCAA
(Reverse)	GTATCCAGTGCGTGTCGTGG
miR-5001-5p	
(Forward)	AGCTGTCGTATCCAGTGCAA
(Reverse)	GTCGTATCCAGTGCGTGTCG
miR-449b-5p	
(Forward)	GCAGTGTATTGTTAGCTGGCG
(Reverse)	TGTCGTGGAGTCGGCAATTG
CRLS1	
(Forward)	AGCAGTCCAGTTAATCTTGGTG
(Reverse)	AGTCTTCCGGCCATAATGATAGT
U6	
(Forward)	GCTTCGGCAGCACATATACTAAAA
(Reverse)	CGCTTCACGAATTTGCGTGTCAT
GAPDH	
(Forward)	CTGGGCTACACTGAGCACC
(Reverse)	AAGTGGTCGTTGAGGGCAATG
