# Supplementary material for: LINC01272 Suppressed Cell Multiplication and Induced Apoptosis Via Regulating MiR-7-5p/CRLS1 Axis in Lung Cancer
Source: J Microbiol Biotechnol. 2021 May 27;31(7):921–32. doi: 10.4014/jmb.2102.02010 (PMC9705921; doi:10.4014/jmb.2102.02010)
Supplement: Supplementary file 1 [file jmb-31-7-921-supple.pdf]

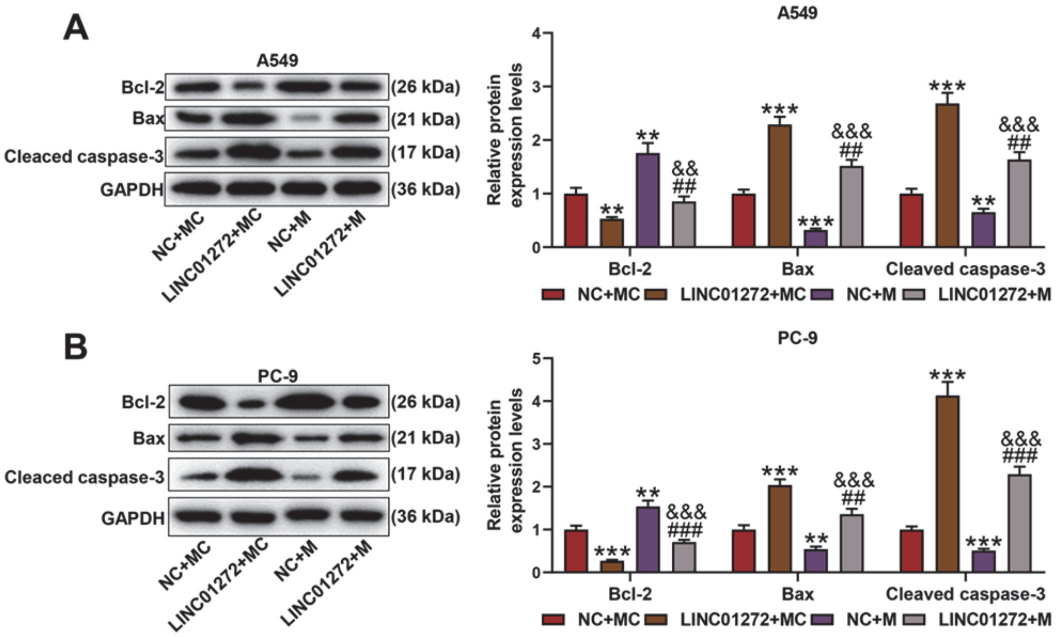
A
A549
Bcl-2
Bax
Cleaced caspase-3
GAPDH
(26 kDa)
(21 kDa)
(17 kDa)
(36 kDa)
NC+MC
LINC01272+MC
NC+M
LINC01272+M
Relative protein expression levels
Bcl-2
Bax
Cleaved caspase-3
B
PC-9

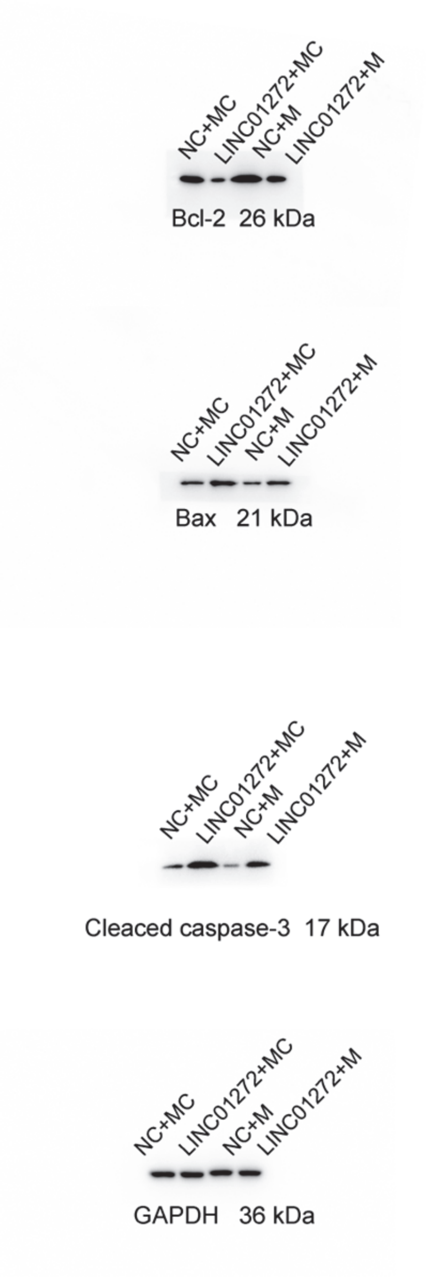

Supplementary figure legends

Supplementary Figure 1.

MiR-7-5p mimic reversed the effect of LINC01272 on levels of apoptosis-related factors in LC cells.

(A and B) Relative Bcl-2, Bax and Cleaved caspase-3 protein expression between of

A549 (A) and PC-9 (B) cells was assessed through Western blot after transfection of miR-7-5p mimic and LINC01272 overexpression plasmid. GAPDH was a loading control. $^{**}p < 0.01$, $^{***}p < 0.001$ vs. NC+MC group; $^{\#\#}p < 0.01$, $^{\#\#\#}p < 0.001$ vs. LINC01272+MC group; $^{\&\&}p < 0.01$, $^{\&\&\&}p < 0.001$ vs. NC+M group. All experiments were repeated independently at least three times. Data was performed as the means ± standard deviation.

Abbreviation: LC, lung cancer; Bcl-2, B-cell lymphoma-2; Bax, Bcl2-Associated X; GAPDH, glyceraldehyde-3-phosphate dehydrogenase; NC, negative control for LINC01272 overexpression plasmid; MC, miR-7-5p mimic control; M, miR-7-5p mimic.
